# Critical care ultrasound evaluation of snuffbox artery vascular tension: a prospective observational study between healthy volunteers and intensive care unit patients

**DOI:** 10.3389/fmed.2026.1722497

**Published:** 2026-04-28

**Authors:** Yao Qin, Yao Zhu, Xiaorong Dai, Wanhong Yin

**Affiliations:** 1 Department of Intensive Care Unit, West China Hospital, Sichuan University, Chengdu, Sichuan, China; 2 Visualized Diagnostics and Therapeutics & Artificial Intelligence Laboratory, Institute of Critical Care Medicine Research, West China Hospital, Sichuan University, Chengdu, Sichuan, China; 3 Department of Critical Care Medicine, Zhongnan Hospital of Wuhan University, Wuhan, Hubei, China; 4 Hubei Clinical Research Center for Critical Care Medicine, Zhongnan Hospital of Wuhan University, Wuhan, Hubei, China

**Keywords:** critical care ultrasound, hemodynamics, microcirculation, reference range, snuffbox artery, vascular tension

## Abstract

**Background:**

Systemic vascular resistance is conventionally assessed using invasive methods; the snuffbox artery resistance index (RI) is a superior non-invasive predictor of lactate clearance in septic shock patients to the perfusion index. However, the normal reference ranges of the snuffbox artery RI and pulsatility index (PI) remain undefined, with inconsistent RI data in healthy populations due to non-standardized measurements.

**Objective:**

This study aimed to establish the normal reference ranges for the snuffbox artery RI and PI in healthy volunteers and to compare these hemodynamic parameters between healthy volunteers and intensive care unit (ICU) patients to evaluate their clinical utility in assessing peripheral microcirculation.

**Design:**

This was a single-center prospective observational cohort study.

**Setting:**

This study was conducted at a 4,900-bed tertiary care hospital with a 215-bed closed ICU in China.

**Participants:**

A total of 91 healthy volunteers—stratified by history of hypertension and age—and 55 ICU patients—classified by shock status—were included in the study from February to November 2024.

**Interventions:**

No interventional measures were implemented; all data were collected through a non-invasive ultrasound and clinical record review.

**Measurements and main results:**

Ultrasound parameters and vital signs were measured under standardized conditions. The normal RI and PI ranges in healthy volunteers were 0.72–0.75 and 1.93–2.03, respectively. Significant differences in the RI and end-diastolic flow velocity (EDV) were found between healthy volunteers and ICU patients (all *p* < 0.001), with no differences in the peak systolic flow velocity (PSV) or PI. EDV was associated with 28-day mortality in patients (*p* < 0.05). Volunteers had significant post-exercise EDV changes, and hypertension/age had no effect on their vascular tension parameters. After excluding 5 patients, the remaining 50 shock and non-shock ICU patients showed no significant differences in all snuffbox artery parameters.

**Conclusion:**

This study established standardized normal reference ranges for the snuffbox artery RI and PI in healthy adults and found significant RI and EDV differences between ICU patients and healthy volunteers. EDV may serve as a potential prognostic indicator, and alterations in diastolic flow may reflect early peripheral vascular tension changes. The lack of parameter differences between shock and non-shock ICU patients reveals the physiological limitations of the snuffbox artery RI as a surrogate for systemic vascular resistance.

## 1 Introduction

Microcirculation is the final critical link in hemodynamic therapy and serves as a key barrier to maintaining cellular oxygen metabolism; improving microcirculatory perfusion is essential for preserving organ perfusion and function (12). Fluid overload resulting from over-optimization of macrocirculation or excessive vasopressor use can induce microcirculatory dysfunction, leading to abnormal cellular oxygen metabolism (1). Hyperpnea can significantly reduce peripheral perfusion and exacerbate hypoperfusion in visceral organs, including the intestines, spleen, and kidneys (2). Even healthy college students have been found to exhibit inadequate microcirculatory perfusion in some studies (3).

Vascular tension assessment is a direct reflection of microcirculatory perfusion, with current clinical monitoring methods including sublingual microcirculation monitoring (4), nail fold microcirculation monitoring (5), and vascular occlusion tests (6). With the widespread application of critical care ultrasound in the visual management of shock, standardized ultrasound protocols enable comprehensive hemodynamic monitoring from macrocirculation to microcirculation—among these, snuffbox artery blood flow evaluation is a reliable non-invasive method for assessing microcirculation. Previously, peripheral vascular tension was judged primarily by clinical experience or exclusion; recent studies have demonstrated that detailed monitoring of snuffbox artery spectral patterns, blood flow velocity, RI, and PI can guide vasopressor titration (7) and hemodynamic management, and the snuffbox artery RI has been validated as a tool for guiding shock resuscitation (8).

Traditional vascular tension assessment is mostly indirect, based on calculations from invasive monitoring data, and is susceptible to multiple confounding factors. Ultrasound-based visualization of peripheral vascular spectra can provide a direct reflection of vascular tension; however, prior research on snuffbox artery hemodynamics has two critical limitations: (1) normal reference ranges for the RI and PI in healthy populations are not well established, with the majority of reports citing an RI threshold of < 0.9 derived from small or non-standardized cohorts and (2) existing studies focus predominantly on critically ill populations, with limited head-to-head comparisons between healthy individuals and ICU patients. We therefore designed this prospective cohort study to characterize the snuffbox artery ultrasound features in healthy volunteers and ICU patients, establish standardized normal reference ranges for the snuffbox artery RI and PI, and explore the correlation between snuffbox artery spectral parameters and microcirculatory perfusion, with the goal of providing a non-invasive reference for clinical hemodynamic management.

## 2 Methods

### 2.1 Ethical review

This prospective cohort study was approved by the Ethics Committee of West China Hospital, Sichuan University (Ethics Approval No. 2024[559]), and written informed consent was obtained from all participants or their legal surrogates (for ICU patients with impaired consciousness). A dedicated data and safety monitoring committee oversaw the study, and a steering committee designed the study protocol to ensure data accuracy and consistency.

### 2.2 Study design and participant selection

#### 2.2.1 Healthy volunteers

The inclusion criteria included individuals who were aged ≥18 years, who were able to complete the study procedures (including exercise tests), and who were able to provide informed consent.

The exclusion criteria included individuals who were aged >70 years, who were obese (body mass index [BMI] ≥ 30 kg/m^2^), who were pregnant or lactating, who had a history of gastrointestinal surgery or chronic gastrointestinal disease, and who had any systemic disease other than essential hypertension (e.g., cardiovascular disease, diabetes, renal/hepatic insufficiency, and peripheral vascular disease).

Rationale for excluding gastrointestinal disease/surgery: Chronic gastrointestinal disease or postoperative status may be associated with systemic low-grade inflammation, microcirculatory dysfunction, or malabsorption-induced vascular endothelial dysfunction—all of which can interfere with snuffbox artery vascular tension measurements. Excluding these individuals ensures the baseline homogeneity of the healthy volunteer group and eliminates confounding factors for microcirculation assessment.

#### 2.2.2 ICU patients

The inclusion criteria included patients who were admitted to the closed ICU of West China Hospital (215 beds, affiliated with a 4,900-bed tertiary hospital), who were aged ≥18 years, and who were hospitalized for ≥24 h.

The exclusion criteria included patients who had poor ultrasound image quality of the snuffbox artery (inadequate Doppler spectral signals), who had incomplete clinical and therapeutic data, and who had withdrawn life-sustaining treatment within 6 h of admission.

*Additional patient characterization*: All patients were assessed for baseline diseases (sepsis, acute respiratory distress syndrome, heart failure, severe trauma, and postoperative complications), disease severity (Acute Physiology and Chronic Health Evaluation II [APACHE II] score within 24 h of admission), shock type (septic, cardiogenic, hypovolemic, and distributive), and shock status according to the international clinical guidelines (presence of hypotension [mean arterial pressure (MAP) < 65 mmHg], tissue hypoperfusion, or hyperlactatemia [lactate > 2 mmol/L]).

### 2.3 Grouping strategy

Healthy volunteers were divided into a hypertensive group (*n* = 17, with a confirmed diagnosis of essential hypertension and stable blood pressure control) and a non-hypertensive group (*n* = 74). The non-hypertensive group was further stratified by age into a young group (≤40 years, *n* = 51) and a middle-aged/elderly group (>40 years, *n* = 23).

A total of 55 ICU patients were initially enrolled in the study, and 5 were excluded based on the above exclusion criteria. The remaining 50 patients were divided into a shock group (*n* = 22, meeting clinical shock criteria) and a non-shock group (*n* = 28) based on hemodynamic status at admission or within 6 h of shock onset.

### 2.4 Data collection

#### 2.4.1 General conditions

All data were collected under standardized conditions: ambient temperature at 24 °C, participants in a calm emotional state, and upper limbs relaxed and placed in a neutral position (sitting or supine). For volunteers, data were collected at rest and at two time points after exercise; for ICU patients, data were collected on admission (non-shock patients) or within 6 h of shock onset (shock patients).

#### 2.4.2 Ultrasound examination protocol

*Unified limb selection*: The non-dominant upper limb was uniformly used for snuffbox artery ultrasound in all participants (the left limb for right-handed individuals and the right limb for left-handed individuals) to eliminate the influence of limb dominance on blood flow parameters.

*Instruments and probes*: Three ultrasound machines were used (Mindray M9, Philips CX50, and Wisonic Clivia). A linear-array probe was used for cardiac function assessment (left ventricular ejection fraction [LVEF]), and a 7–12 MHz linear-array probe was used for snuffbox artery vascular imaging.

*Scanning method*: The linear-array probe was placed over the snuffbox area, identified by the pulsation of the radial artery between the extensor pollicis tendons and the distal radius. Color Doppler mode was used for imaging, with fixed gain and wall filter settings to ensure consistency. Doppler spectra, blood flow images, and mean flow velocity were recorded; the RI and PI were automatically calculated using the ultrasound machine. Three consecutive measurements were taken for each parameter, and the average value was used for statistical analysis.

*Personnel qualification*: All ultrasound examinations were performed by two or more attending physicians with certified critical care ultrasound qualifications and at least 5 years of clinical experience in critical care ultrasound.

#### 2.4.3 Exercise test for volunteers

Exercise was defined as a combination of running, deep squats, and stair climbing until the heart rate (HR) reached 120 bpm; the heart rate was maintained at this level for 5 min, and then ultrasound and vital sign data were collected immediately.

#### 2.4.4 Clinical and therapeutic data collection for patients

Relevant therapeutic measures were recorded in detail for all patients during ultrasound measurement, including vasopressor use (type: norepinephrine, metaraminol, and pituitrin), mechanical ventilation, and laboratory indicators (lactate) within 1 h of ultrasound measurement.

### 2.5 Outcome measures

The outcome measures include the following:

1. Baseline demographic and vital sign data: age, sex, BMI, heart rate (HR), systolic blood pressure (SBP), diastolic blood pressure (DBP), and LVEF;
2. Snuffbox artery ultrasound parameters: RI, PI, PSV, and EDV;
3. 28-day mortality of ICU patients;
4. Changes in snuffbox artery parameters and vital signs in volunteers before and after exercise;
5. Clinical characteristics of shock patients: shock type, APACHE II score, vasopressor/inotrope dosage, lactate level, and mechanical ventilation status.

### 2.6 Statistical analysis

Categorical variables were presented as counts and percentages and compared using the chi-squared test or Fisher’s exact test. Continuous variables were tested for normality. Normally distributed variables were presented as mean ± standard deviation (SD) and compared using the independent samples t-test or a one-way analysis of variance (ANOVA). Non-normally distributed variables were presented as median (interquartile range [IQR]) and compared using the Mann–Whitney U-test or the Kruskal–Wallis H test. A correlation analysis was performed between EDV and 28-day mortality using the Cox proportional hazards model. A two-sided *p*-value of < 0.05 was considered statistically significant. All statistical analyses were performed using SPSS 27.0 and GraphPad Prism 10.0 software.

For the snuffbox artery resistance index (RI) and pulsatility index (PI), the 95% confidence interval (CI) of the mean was used to present the normal reference range instead of the mean ± standard deviation (SD). This is because the 95% CI of the mean reflects the precision of the population parameter estimate, which is more clinically relevant for establishing a normative reference range. In contrast, SD only represents the variability within the study sample and cannot accurately reflect the true range of the population.

## 3 Results

### 3.1 Study population baseline characteristics

**Figure 1:**
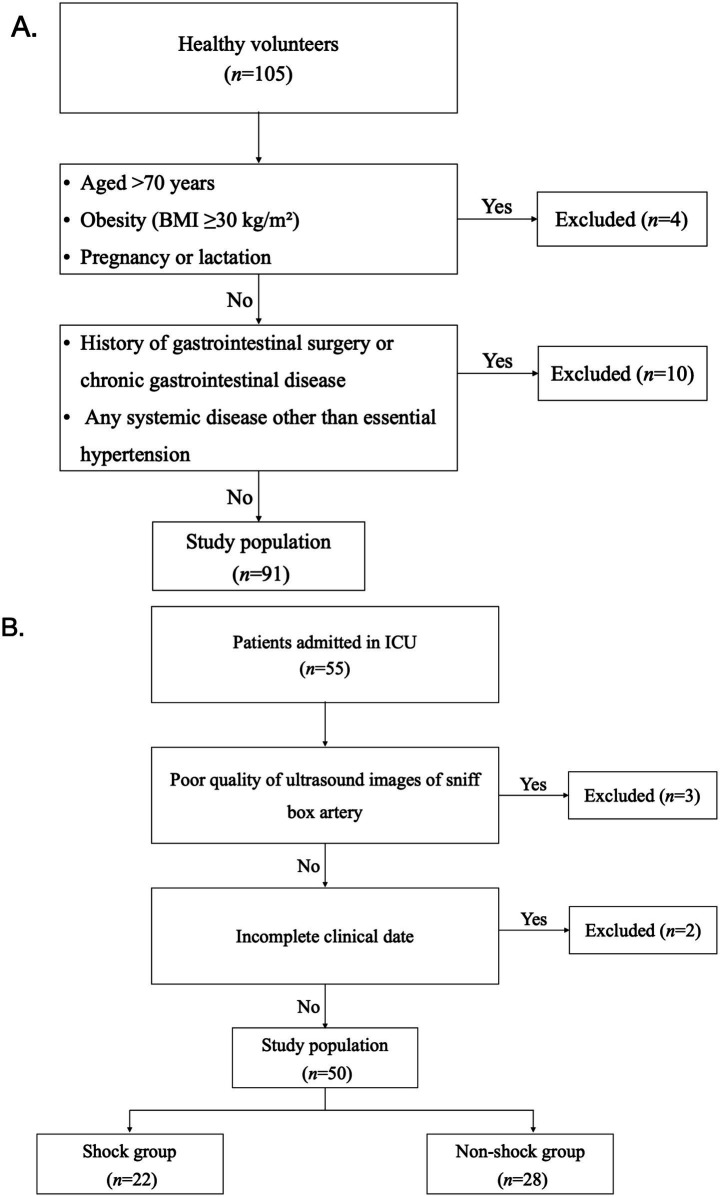
Flow diagram of the study population screening and enrollment. **(A)** Flow diagram of healthy volunteer screening and enrollment (initial recruitment: *n* = 105; excluded: *n* = 14; final study population: *n* = 91). **(B)** Flow diagram of ICU patient enrollment and subgroup allocation (initial admission: *n* = 55; excluded: *n* = 5; final study population: *n* = 50; divided into a shock group [*n* = 22] and a non-shock group [*n* = 28]).

A total of 91 healthy volunteers and 55 ICU patients were initially enrolled. Among the ICU patients, 5 were excluded—3 with poor ultrasound image quality and 2 with incomplete therapeutic data, resulting in 50 patients for the final shock subgroup analysis. Detailed flow diagrams of healthy volunteer screening and ICU patient enrollment are presented in Figures 1A,B, respectively. Baseline demographic and vital sign differences between the 91 volunteers and the 55 initially enrolled ICU patients were statistically significant for age, BMI, gender composition, SBP, and DBP (all *p* < 0.05), whereas the HR showed no significant difference (*p* = 0.168) (Table 1).

**Table 1:** Baseline data of healthy volunteers and initially enrolled ICU patients.

Characteristics	Volunteers (*n* = 91)	ICU patients (*n* = 55)	*p*-value
Age, years (SD)	37 (12.04)	55 (14.56)	<0.001
BMI, kg/m^2^ (SD)	22.98 (4.71)	24.58 (2.49)	0.020
Sex, *n* (%)			<0.001
Male	43 (47.3)	43 (78.2)	
Female	48 (52.7)	12 (21.8)	
HR, bpm (IQR)	81 (74–90)	86 (73–98)	0.168
SBP, mmHg (IQR)	120 (112–129)	130 (123–140)	<0.001
DBP, mmHg (IQR)	76 (70–84)	71 (62–79)	0.009
Snuffbox artery RI (SD)	0.73 (0.04)	0.85 (0.11)	<0.001
Snuffbox artery PI (SD)	1.98 (0.12)	2.05 (0.23)	0.124
Snuffbox artery PSV, cm/s (IQR)	42.6 (35.8–51.2)	45.3 (38.1–53.7)	0.493
Snuffbox artery EDV, cm/s (IQR)	9.8 (7.6–12.3)	6.5 (4.2–8.9)	<0.001

### 3.2 Clinical characteristics and snuffbox artery ultrasound parameters of shock and non-shock ICU patients

Among the 50 ICU patients finally included, 22 were in the shock group and 28 in the non-shock group (Table 2). The shock subgroup was predominantly composed of septic shock (81.8%), followed by hypovolemic shock (13.6%) and cardiogenic shock (4.6%). Compared with the non-shock group, the shock group had significantly higher APACHE II scores, lactate levels, norepinephrine (NE) dosage, and 28-day mortality (all *p* < 0.05). Regarding additional vasopressors, 6 (27.3%) patients in the shock group received pituitrin, with a median dosage of 1.32 (IQR: 0.86–1.88) U/h, whereas no patients in the non-shock group used pituitrin (*p* < 0.001). A total of 8 (36.4%) patients in the shock group received metaraminol, with a median dosage of 2.05 (IQR: 1.21–2.89) mg/h, compared to only 1 (3.6%) patient in the non-shock group (*p* = 0.003).

**Table 2:** Clinical characteristics and snuffbox artery ultrasound parameters of shock and non-shock ICU patients (*n* = 50).

Characteristics	Shock group (*n* = 22)	Non-shock group (*n* = 28)	*p*-value
Age, years (IQR)	57 (45–69)	53 (41–65)	0.356
Male, *n* (%)	18 (81.8)	21 (75.0)	0.542
BMI, kg/m^2^ (SD)	24.8 (2.7)	24.3 (2.3)	0.489
APACHE II score (IQR)	22 (18–26)	14 (10–18)	<0.001
Shock type, *n* (%)			
Septic shock	18 (81.8)	-	-
Hypovolemic shock	3 (13.6)	-	-
Cardiogenic shock	1 (4.6)	-	-
Hypertension history, *n* (%)	5 (22.7)	7 (25.0)	0.831
NE dosage, μg/kg/min (IQR)	0.3 (0.1–0.5)	-	<0.001
Pituitrin use, *n* (%)	6 (27.3)	-	<0.001
Pituitrin dosage, U/h (IQR)	1.32 (0.86–1.88)	-	<0.001
Metaraminol use, *n* (%)	8 (36.4)	1 (3.6)	0.003
Metaraminol dosage, mg/h (IQR)	2.05 (1.21–2.89)	1.80 (−)	0.005
Lactate, mmol/L (IQR)	3.8 (2.5–5.2)	1.6 (1.1–2.3)	<0.001
Mechanical ventilation, *n* (%)	19 (86.4)	12 (42.9)	<0.001
Snuffbox artery RI (SD)	0.86 (0.10)	0.84 (0.12)	0.988
Snuffbox artery PI (SD)	2.07 (0.21)	2.03 (0.24)	0.902
Snuffbox artery PSV, cm/s (IQR)	44.7 (37.5–52.3)	46.1 (39.2–55.1)	0.071
Snuffbox artery EDV, cm/s (IQR)	6.3 (4.0–8.7)	6.8 (4.5–9.2)	0.262
28-day mortality, *n* (%)	7 (31.8)	2 (7.1)	0.019

**Figure 2:**
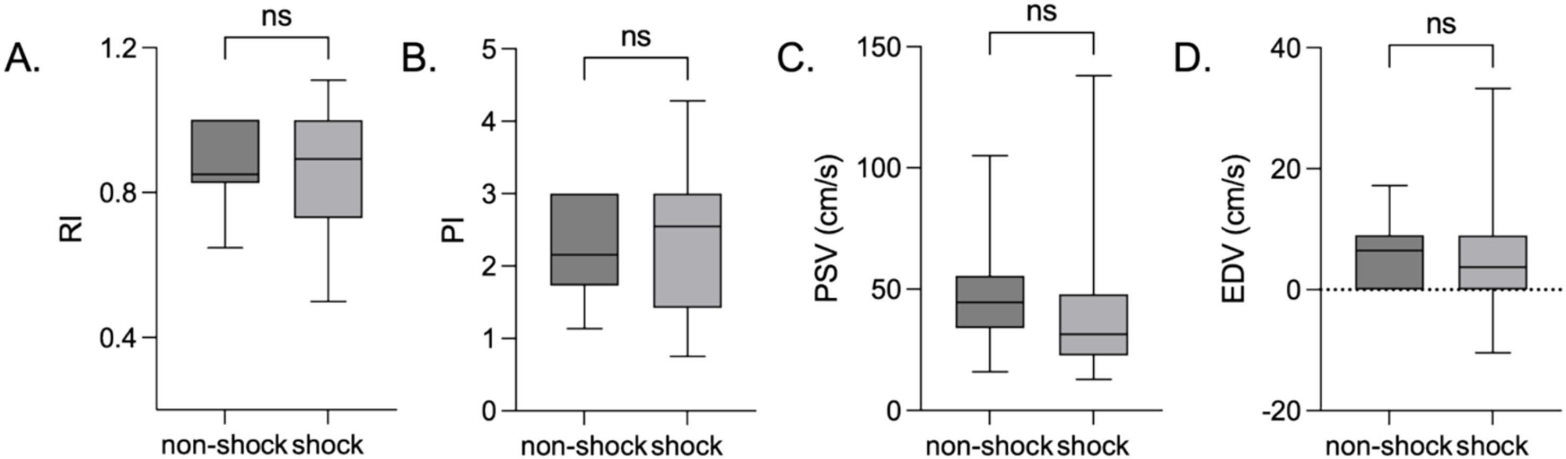
Snuffbox artery ultrasound parameters in shock and non-shock ICU patients. ****p < 0.0001; ***p < 0.001; **p < 0.01; *p < 0.05; ns *p* > 0.05. **(A)** RI; **(B)** PI; **(C)** PSV; **(D)** EDV.

However, no statistically significant differences in the snuffbox artery RI (*p* = 0.988), PI (*p* = 0.902), PSV (*p* = 0.071), or EDV (*p* = 0.262) were found between the shock (*n* = 22) and non-shock (*n* = 28) patient groups (Figure 2). EDV was negatively correlated with 28-day mortality in the overall patient group (HR = 0.89, 95%CI: 0.81–0.98, *p* < 0.05).

### 3.3 Comparison of snuffbox artery ultrasound parameters between volunteers and initially enrolled ICU patients

**Figure 3:**
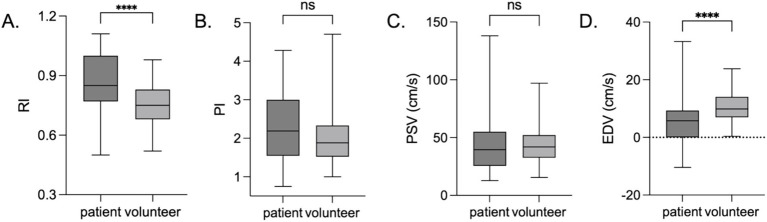
Snuffbox artery ultrasound parameters in volunteers and ICU patients. ****p < 0.0001; ***p < 0.001; **p < 0.01; *p < 0.05; ns *p >* 0.05. **(A)** RI; **(B)** PI; **(C)** PSV; **(D)** EDV.

Significant differences were observed in the snuffbox artery RI (*p* < 0.001) and EDV (*p* < 0.001) between the volunteer and ICU patient groups. No statistically significant differences were found in the PSV (*p* = 0.493) or PI (*p* = 0.124) (Figure 3). EDV was significantly lower in patients than in volunteers, whereas the RI was significantly higher in patients than in volunteers. The normal reference ranges of the snuffbox artery RI and PI in healthy volunteers were established as 0.72–0.75 and 1.93–2.03, respectively, based on the 95% confidence interval of the measured values.

To eliminate the confounding effects of age and body mass index (BMI) on the comparison of snuffbox artery parameters between healthy volunteers and ICU patients, a multivariate linear regression analysis was performed with RI and EDV as dependent variables and age and BMI as covariates. After adjusting for age and BMI, the differences in snuffbox artery RI (*β* = 0.326, 95%CI: 0.215–0.437, *p* < 0.001) and EDV (*β* = −0.289, 95%CI: −0.402 to −0.176, *p* < 0.001) between the two groups remained statistically significant. This indicated that the significant differences in RI and EDV between healthy volunteers and ICU patients were independent of age and BMI, and the main influencing factor was the pathological state of critical illness.

### 3.4 Comparison of snuffbox artery parameters between hypertensive and non-hypertensive volunteers

**Figure 4:**
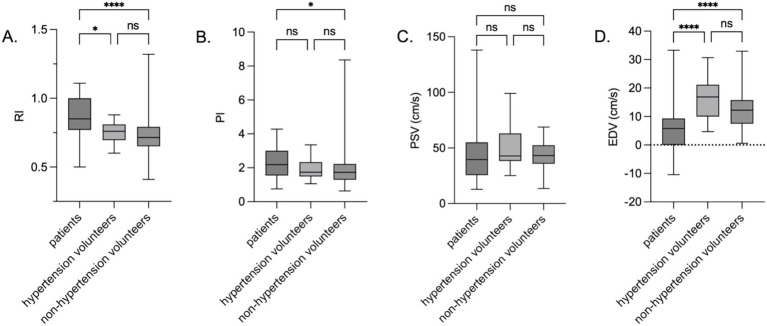
Snuffbox artery ultrasound parameters in the patient group, hypertensive volunteer group, and non-hypertensive volunteer group. ****p < 0.0001; ***p < 0.001; **p < 0.01; *p < 0.05; ns *p* > 0.05. **(A)** RI; **(B)** PI; **(C)** PSV; **(D)** EDV.

Baseline data of the hypertensive (*n* = 17, mean age: 48 years) and non-hypertensive (*n* = 74, mean age: 33 years) volunteer groups differed significantly in age, BMI, HR, and SBP (all *p* < 0.05), with no differences in the composition of DBP or gender (*p* > 0.05) (Table 3). No statistically significant differences in the snuffbox artery RI, PI, PSV, or EDV were found between the hypertensive and non-hypertensive groups (all *p* > 0.05) (Figure 4). In contrast, compared with both volunteer subgroups, patients had significantly higher RI and lower EDV (*p* < 0.001), and patients had a significantly higher PI than the hypertensive volunteer subgroup (*p* < 0.05); PSV showed no significant difference among the three groups (*p* > 0.05).

**Table 3:** Baseline data of hypertensive and non-hypertensive volunteers.

Characteristics	Hypertension (*n* = 17)	Non-hypertension (*n* = 74)	*p*-value
Age, years (IQR)	48 (36.5–53)	33 (26–48)	0.002
BMI, kg/m_2_	24.97 (23.78–27.23)	21.82 (19.94–24.43)	<0.001
Sex *n*, %			0.986
Male	8 (47.1)	35 (47.3)	
Female	9 (52.9)	39 (52.7)	
HR, bpm (SD)	87.47 (22.29)	76 (11.49)	0.001
SBP, mmHg (IQR)	142 (139–155)	117.5 (111–123)	<0.001
DBP, mmHg (SD)	83 (10.77)	73.35 (8.97)	0.830

### 3.5 Comparison of snuffbox artery parameters between young and middle-aged/elderly non-hypertensive volunteers

**Figure 5:**
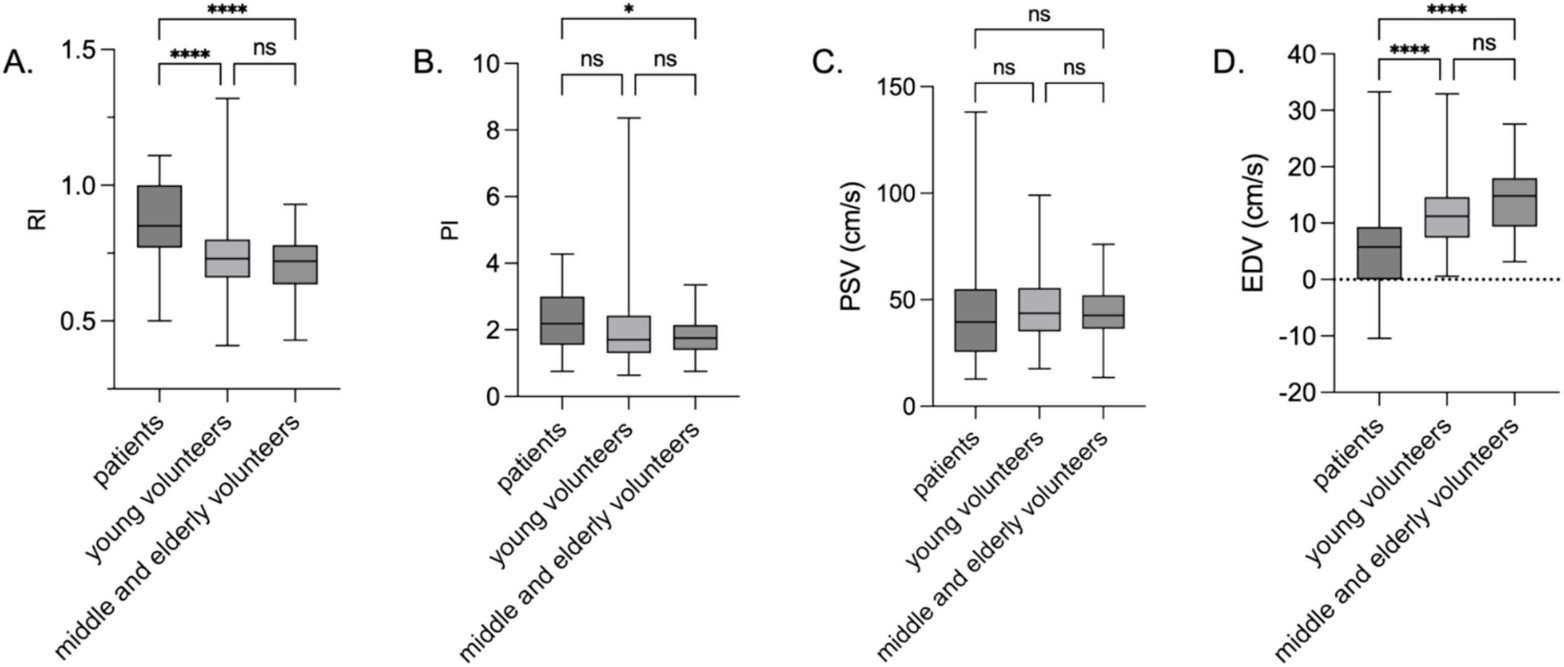
Snuffbox artery ultrasound parameters in the patient group, the young volunteer group, and the middle-aged/elderly volunteer group. ****p < 0.0001; ***p < 0.001; **p < 0.01; *p < 0.05; ns *p >* 0.05. **(A)** RI; **(B)** PI; **(C)** PSV; **(D)** EDV.

The non-hypertensive volunteer group was stratified into a young group (≤40 years, *n* = 51) and a middle-aged/elderly group (>40 years, *n* = 23). Baseline BMI, SBP, and DBP differed significantly between the two subgroups (all *p* < 0.05), with no differences in the gender composition or HR (*p* > 0.05) (Table 4). No significant differences in the snuffbox artery RI, PI, PSV, or EDV were found between the young and middle-aged/elderly volunteer subgroups (all *p* > 0.05). Compared with both volunteer subgroups, patients had significantly higher RI and PI and lower EDV (all *p* < 0.001); PSV showed no significant difference among the three groups (*p* > 0.05) (Figure 5).

**Table 4:** Baseline data of young and middle-aged/elderly non-hypertensive volunteers.

Characteristics	Youth group (*n* = 51)	Middle and elderly group (*n* = 23)	*p*-value
Age, years (IQR)	28 (24–33)	50 (48–52.5)	<0.001
BMI, kg/m_2_	20.62 (19.36–23.04)	24.42 (23.42–25.93)	<0.001
Sex *n*, %			0.196
Male	29 (52.7)	14 (38.9)	
Female	26 (47.3)	9 (61.1)	
SBP, mmHg (IQR)	114 (106–123)	128.5 (120–141.75)	<0.001
DBP, mmHg (IQR)	72 (66–79)	81.5 (76.25–90)	<0.001
HR, bpm (SD)	82 (10.87)	84 (18.24)	0.553

### 3.6 Comparison of snuffbox artery parameters between the left and right upper limbs in volunteers

**Figure 6:**
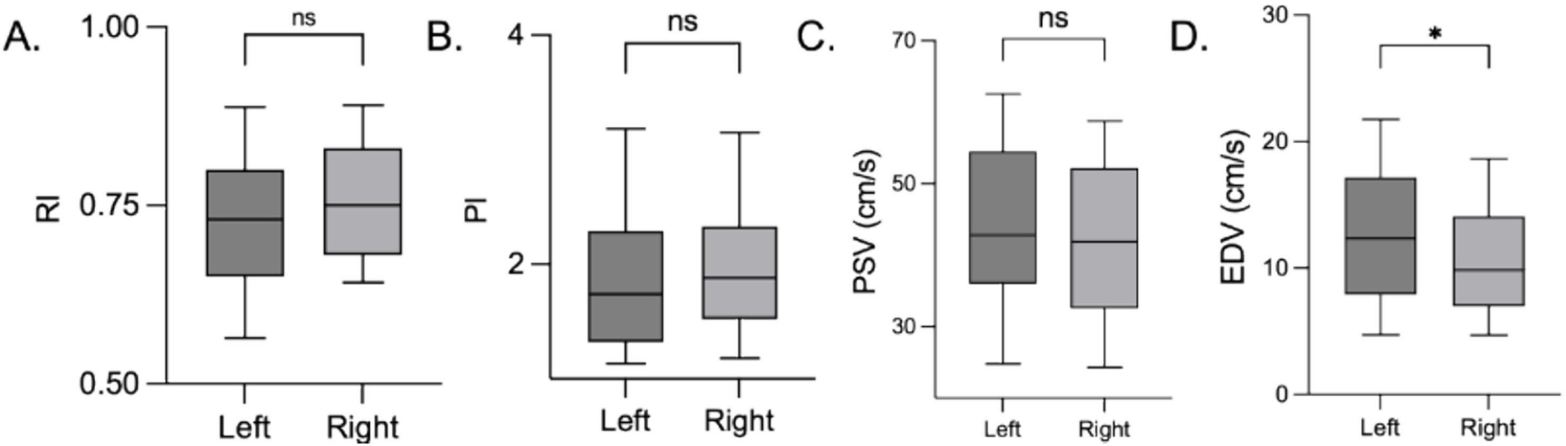
Snuffbox artery ultrasound parameters in the left and right upper limbs of healthy volunteers. ****p < 0.0001; ***p < 0.001; **p < 0.01; *p < 0.05; ns *p* > 0.05. **(A)** RI; **(B)** PI; **(C)** PSV; **(D)** EDV.

For healthy volunteers, EDV showed a statistically significant difference between the left and right snuffbox arteries (95%CI: −3.91 to −0.41, *p* = 0.01), with no significant differences in RI (*p* = 0.104), PI (*p* = 0.156), or PSV (*p* = 0.256) (Figure 6).

### 3.7 Changes in snuffbox artery parameters in volunteers before and after exercise

#### 3.7.1 Hypertensive vs. non-hypertensive volunteers

In the non-hypertensive group, HR, cardiac output (CO), RI, and EDV changed significantly after exercise (all *p* < 0.05). In the hypertensive group, HR, right snuffbox artery PSV, and right snuffbox artery EDV showed significant post-exercise changes (all *p* < 0.05). After exercise, the non-hypertensive group exhibited a downward trend in RI and PI and an upward trend in PSV and EDV; the hypertensive group showed a similar trend in all parameters. Post-exercise, only the right snuffbox artery EDV differed significantly between the two groups (*p* = 0.045).

#### 3.7.2 Young vs. middle-aged/elderly non-hypertensive volunteers

In the young group, HR, CO, and right snuffbox artery RI changed significantly after exercise (all *p* < 0.05). In the middle-aged/elderly group, HR, CO, right snuffbox artery PSV, and right snuffbox artery EDV showed significant post-exercise changes (all *p* < 0.05). Both subgroups exhibited a downward trend in RI and PI and an upward trend in EDV after exercise. Post-exercise, the right snuffbox artery PI (*p* = 0.046) and the left snuffbox artery EDV (*p* = 0.037) differed significantly between the two subgroups.

## 4 Discussion

Snuffbox artery spectral characterization is an important non-invasive method for evaluating peripheral microcirculatory perfusion, and optimizing microcirculatory perfusion is a core goal of hemodynamic therapy for critically ill patients (13, 14). Previous studies have confirmed that the snuffbox artery RI (SBRI) is a better indicator of tissue hypoperfusion than the cardiac index (CI) in septic shock patients and can effectively predict lactate clearance (8). However, the normal reference ranges of snuffbox artery hemodynamic parameters in healthy individuals have long been undefined, with the majority of prior reports citing a qualitative RI threshold of < 0.9 (9), and no standardized quantitative range for RI or PI having been established to date. This study addresses this critical gap and represents the first prospective study to establish standardized normal reference ranges of the snuffbox artery RI (0.72–0.75) and PI (1.93–2.03) in healthy adults under controlled, standardized measurement conditions.

### 4.1 Normal snuffbox artery RI values: comparison with prior literature and explanations for discrepancies

The most significant finding of this study is the establishment of a normal snuffbox artery RI range of 0.72–0.75 in healthy volunteers, which is substantially lower than the previously reported threshold of < 0.9 (9) and the RI values of 0.9–1.1 reported in some clinical studies (7). Two key methodological and population-based factors explain this discrepancy:

1. *Study population heterogeneity*: The vast majority of prior studies on the snuffbox artery RI were conducted on critically ill or septic populations (7, 8), where systemic inflammation, sympathetic hyperactivity, and microcirculatory dysfunction lead to elevated peripheral vascular resistance and thus higher RI values. In contrast, the healthy volunteers in this study were enrolled with strict exclusion criteria (excluding peripheral vascular disease, systemic inflammation, and the majority of chronic diseases), ensuring a truly normal cohort with unimpaired microcirculation.
2. *Standardized measurement protocols*: This study implemented strict standardization of ultrasound measurements (unified non-dominant limb selection, fixed ambient temperature, neutral limb position, and three consecutive measurements for averaging), whereas prior studies often used non-standardized methods (e.g., random limb selection, no control of environmental conditions, and single measurements). Limb dominance is a critical confounding factor, as the dominant limb has higher basal blood flow and vascular tone (10), and non-standardized measurements can lead to the overestimation of RI values.
3. *Elimination of confounding clinical factors*: Prior studies on “healthy” cohorts often included individuals with unmanaged hypertension, obesity, or subclinical vascular disease—all of which can increase peripheral vascular resistance. This study stratified volunteers according to hypertension history and found no significant effect of well-controlled essential hypertension on snuffbox artery RI, confirming that the established normal range is robust to mild, controlled chronic conditions.

Physiologically, the RI range of 0.72–0.75 in healthy volunteers indicates the normal state of peripheral vascular tone: the snuffbox artery is a peripheral muscular artery with moderate basal resistance, and the low RI value is consistent with unimpaired microcirculatory perfusion and normal vascular endothelial function. This standardized range provides a critical reference for clinical practice, as an RI value above this range in ICU patients can now be quantitatively defined as abnormal peripheral vascular resistance, rather than relying on the vague qualitative threshold of > 0.9.

### 4.2 Snuffbox artery parameters in ICU patients: differences between healthy volunteers and prognostic value

This study found that ICU patients had significantly higher RI and lower EDV than healthy volunteers, with no differences in PSV or PI. This finding is consistent with the pathophysiology of critical illness: systemic inflammation (e.g., cytokine storm), sympathetic hyperactivity, and inflammatory mediator–induced vasodilatory dysfunction lead to impaired microcirculatory perfusion and increased peripheral vascular resistance (6), resulting in elevated snuffbox artery RI and reduced diastolic blood flow. EDV was further found to be negatively correlated with 28-day mortality in ICU patients (*p* < 0.05), suggesting that snuffbox artery diastolic flow is a potential prognostic indicator for critically ill patients. Diastolic blood flow is a key marker of microcirculatory perfusion, as it reflects the ability of peripheral vessels to maintain blood flow during diastole, an impairment of this ability indicates severe microcirculatory dysfunction and is associated with poor clinical outcomes.

Notably, hypertension history and age had no significant effect on snuffbox artery vascular tension parameters in healthy volunteers, indicating that the snuffbox artery RI and PI have good stability in the healthy adult population and are less affected by age and mild, well-controlled essential hypertension. Exercise induced characteristic changes in snuffbox artery parameters in volunteers: a decrease in RI and PI and an increase in PSV and EDV, with diastolic flow changes being more significant. This is because exercise increases systemic cardiac output and induces peripheral vasodilation (increasing vascular lumen diameter) (10), leading to increased blood flow velocity and reduced vascular resistance. The more significant change in EDV suggests that diastolic flow is more sensitive to early peripheral vascular tension alterations, which is a key finding of this study and provides a new perspective for early microcirculation assessment.

### 4.3 Lack of differences in snuffbox artery parameters between shock and non-shock ICU patients: physiological limitations and contextualization

A key finding of this study is the absence of significant differences in the snuffbox artery RI, PI, PSV, or EDV between shock and non-shock ICU patients (*n* = 50). This result, while seemingly counterintuitive, is consistent with existing literature and reflects the fundamental physiological limitations of using peripheral Doppler-derived RI as a surrogate for systemic vascular resistance (SVR).

First, peripheral arterial RI (including snuffbox artery RI) is a local vascular parameter that reflects regional peripheral vascular resistance, whereas SVR is a global hemodynamic parameter that integrates the resistance of the entire systemic vasculature. A recent cross-sectional study (11) found a weak correlation between the radial artery RI (a close surrogate for snuffbox artery RI) and SVR index (r = 0.23, *p* < 0.05), confirming that local peripheral RI cannot fully represent global SVR. In ICU patients, even non-shock patients have varying degrees of systemic inflammation, organ dysfunction, or vasopressor exposure—all of which increase local peripheral vascular resistance and reduce the hemodynamic difference between the shock and non-shock groups.

Second, the heterogeneity of shock states and ICU patient characteristics further limits the predictive value of the snuffbox artery RI. The shock subgroup in this study was predominantly septic shock (81.8%), a condition characterized by distributive vasodilation and heterogeneous microcirculatory perfusion: while global SVR is reduced, regional peripheral vascular resistance can be increased due to local vasoconstriction and microcirculatory shunting (8). Non-shock ICU patients, on the other hand, often receive vasopressor therapy for hemodynamic support, which can increase local peripheral vascular resistance and elevate RI values to levels comparable to those in shock patients. This “hemodynamic overlap” between the two groups is the primary reason for the lack of significant differences in snuffbox artery parameters.

Third, this study only collected single time-point measurements of snuffbox artery parameters, while peripheral vascular tension is a dynamic parameter that changes with therapeutic interventions (e.g., vasopressor titration and fluid resuscitation) and disease progression. A single measurement cannot capture the dynamic changes of peripheral microcirculation, and serial measurements would likely reveal differences between shock and non-shock patients that were not observed in this study.

### 4.4 Clinical implications of the study

This study has two key clinical implications for critical care ultrasound practice:

1. *Standardized normal reference ranges*: The establishment of the snuffbox artery RI (0.72–0.75) and PI (1.93–2.03) provides a quantitative reference for clinical assessment—ICU patients with an RI exceeding this range can be identified as having abnormal peripheral vascular resistance, enabling early intervention for microcirculatory dysfunction.
2. *EDV as a prognostic indicator*: The finding that EDV is negatively correlated with 28-day mortality suggests that snuffbox artery diastolic flow can be used as a simple, non-invasive prognostic marker for ICU patients, complementing existing clinical and laboratory indicators (e.g., lactate and APACHE II score).
3. *Caution in using RI for SVR assessment*: The lack of differences between shock and non-shock patients highlights that the snuffbox artery RI should not be used in isolation as a surrogate for SVR. Instead, it should be combined with other hemodynamic parameters (e.g., cardiac output and MAP) and clinical data for a comprehensive assessment of peripheral microcirculation.

### 4.5 Study limitations

1. This is a single-center study with a relatively small sample size, particularly for the hypertensive volunteer subgroup (*n* = 17) and the shock patient group (*n* = 22); the results need to be verified by multi-center, large-sample studies.
2. Among the 55 initially enrolled ICU patients, 5 were excluded for shock subgroup analysis due to poor ultrasound image quality and incomplete clinical data, which may introduce selection bias; future studies should optimize ultrasound acquisition protocols and clinical data collection to minimize exclusion rates.
3. Only a single time point of ultrasound data was collected for ICU patients, without dynamic monitoring of parameter changes in response to therapeutic interventions or disease progression. Subsequent studies should conduct serial measurements to explore the dynamic changes of snuffbox artery parameters in critically ill patients.
4. This study did not analyze correlations between snuffbox artery parameters and other microcirculation assessment indicators (e.g., sublingual microcirculation and mottling score). Subsequent studies should use multi-modal microcirculation assessments to validate the clinical value of snuffbox artery ultrasound.
5. The exercise test for volunteers employed a combined exercise mode, so the effect of a single exercise type on snuffbox artery parameters was not determined. Follow-up studies could stratify by exercise type to clarify the relationship between exercise and peripheral vascular tension.
6. The shock subgroup was predominantly septic shock, with only a few hypovolemic and cardiogenic shock patients. Therefore, the results may not be generalizable to other shock types, and future studies should enroll a more heterogeneous shock population.
7. Although healthy volunteers and ICU patients had significant differences in age, gender, and BMI (all *p* < 0.05), the primary comparison of RI and EDV was initially conducted without adjusting for these confounders. However, a subsequent sensitivity analysis using a multivariate linear regression analysis confirmed that the differences in RI and EDV between the two groups remained statistically significant after adjusting for age and BMI. However, gender was not further analyzed due to uneven distribution between groups, which may represent a potential limitation.

## 5 Conclusion

This study established standardized normal reference ranges for the snuffbox artery RI (0.72–0.75) and PI (1.93–2.03) in healthy adults under controlled measurement conditions, addressing the long-standing lack of quantitative reference values in the field. It also identified significant differences in snuffbox artery RI and EDV between ICU patients and healthy volunteers and found that EDV is negatively correlated with 28-day mortality in ICU patients, highlighting its potential as a non-invasive prognostic indicator. Hypertension history and age had no significant effect on snuffbox artery vascular tension parameters in healthy volunteers, and exercise-induced diastolic flow alterations may indicate early peripheral vascular tension changes. The absence of hemodynamic differences between shock and non-shock ICU patients highlights the physiological limitations of snuffbox artery RI as a surrogate for systemic vascular resistance, emphasizing that it should be used in combination with other hemodynamic and clinical data for comprehensive microcirculation assessment. Overall, snuffbox artery ultrasound is a reliable, non-invasive method for evaluating peripheral microcirculation, and the standardized reference ranges and measurement protocol provided in this study lay a foundation for its clinical application in critical care hemodynamic management.

## Data Availability

The original contributions presented in the study are included in the article/Supplementary material, further inquiries can be directed to the corresponding author.
