# Supplementary material for: Critical care ultrasound evaluation of snuffbox artery vascular tension: a prospective observational study between healthy volunteers and intensive care unit patients
Source: Front Med (Lausanne). 2026 Apr 28;13:1722497. doi: 10.3389/fmed.2026.1722497 (PMC13160889; doi:10.3389/fmed.2026.1722497)
Supplement: Supplementary file 1 [file Supplementary_file_1.docx]

|  | hypertension(n=17) | |  | non-hypertension(n=74) | |  |
| --- | --- | --- | --- | --- | --- | --- |
| characteristic | before exercise | after exercise | P | before exercise | after exercise | P |
| HR, bpm | 87 (22) | 118 (25) | 0.001 | 81 (11) | 110 (17) | <0.001 |
| CO, L/min | 4.89 (2.41) | 6.22 (2.82) | 0.149 | 3.63(2.83,4.61) | 4.91(3.47,5.45) | <0.001 |
| Left RI, | 0.75 (0.08) | 0.69 (0.14) | 0.147 | 0.72 (0.15) | 0.70 (0.14) | 0.317 |
| Right RI, | 0.73 (0.11) | 0.74 (0.09) | 0.737 | 0.76 (0.10) | 0.71 (0.12) | 0.020 |
| Left PI, | 1.89 (0.61) | 1.58 (0.43) | 0.099 | 1.72(1.29,2.23) | 1.58(1.24,1.95) | 0.311 |
| Right PI, | 1.89(1.43,2.24) | 1.86(1.55,2.43) | 0.100 | 1.86(1.52,2.42) | 1.73(1.33,2.15) | 0.186 |
| Left PSV, cm/s | 42.78(38.29,63.11) | 43.28(27.42,55.56) | 0.394 | 43.30 (13.07) | 43.83 (16.87) | 0.830 |
| Right PSV, cm/s | 42.53(34.13,47.09) | 53.45(39.57,65.30) | 0.045 | 41.76(32.33,53.15) | 45.99(38.82,56.14) | 0.068 |
| Left EDV, cm/s | 15.98 (7.54) | 17.10 (7.94) | 0.677 | 12.21(7.49,15.79) | 13.29(6.96,18.42) | 0.249 |
| Right EDV, cm/s | 10.12(6.87,18.97) | 21.27(12.15,25.72) | 0.040 | 9.77(7.33,13.88) | 13.52(8.39,18.26) | 0.04 |

Supplementary Table 1. Comparison of ultrasound vascular tension parameters of hypertensive and non-hypertensive group before and after exercise. Normally distributed variables are presented as mean (SD); non-normally distributed variables are presented as median (IQR).

| characteristic | Hypertension (n=17) | non-hypertension (n=74) | P |
| --- | --- | --- | --- |
| HR, bpm | 118 (25) | 110 (17) | 0.097 |
| CO, L/min | 5.49(4.32,8.49) | 4.91(3.47,5.45) | 0.069 |
| Left RI, | 0.69 (0.14) | 0.70 (0.14) | 0.843 |
| Right RI, | 0.74 (0.09) | 0.71 (0.12) | 0.842 |
| Left PI, | 1.6(1.24,1.89) | 1.58(1.24,1.95) | 0.899 |
| Right PI, | 1.86(1.55,2.43) | 1.75(1.33,2.15) | 0.398 |
| Left PSV, cm/s | 43.28(27.42,55.56) | 43.54(33.83,53.95) | 0.875 |
| Right PSV, cm/s | 53.45(39.57,65.3) | 45.99(38.82,56.14) | 0.209 |
| Left EDV, cm/s | 16.88(9.51,21.17) | 13.29(6.96,18.42) | 0.143 |
| Right EDV, cm/s | 21.27(12.15,25.72) | 13.52(8.39,18.26) | 0.045 |
|  |  |  |  |

Supplementary Table 2. Comparison of ultrasound vascular tension parameters of hypertensive and non-hypertensive group after exercise. Normally distributed variables are presented as mean (SD); non-normally distributed variables are presented as median (IQR).

|  | youth group (n=51) | |  | middle and elderly group (n=23) | |  |
| --- | --- | --- | --- | --- | --- | --- |
| characteristic | before exercise | after exercise | P | before exercise | after exercise | P |
| HR, bpm | 80(75,89) | 110(100,125) | <0.001 | 84 (18) | 109 (20) | <0.001 |
| EF, % | 76.6(71.08,81.52) | 76.96(72.94,82.00) | 0.572 | 68.52 (10.63) | 72.52 (13.26) | 0.166 |
| CO, L/min | 3.75(3.06,4.85) | 4.97(3.57,6.11) | 0.001 | 3.55(2.59,5.37) | 4.77(3.42,5.77) | 0.014 |
| Left RI, | 0.74 (0.14) | 0.70 (0.14) | 0.177 | 0.70 (0.12) | 0.69 (0.14) | 0.741 |
| Right RI, | 0.76 (0.10) | 0.70 (0.11) | 0.008 | 0.75 (0.10) | 0.75 (0.12) | 0.896 |
| Left PI, | 1.70(1.30,2.43) | 1.53(1.22,1.90) | 0.130 | 1.75(1.39,2.14) | 1.72(1.42,1.89) | 0.804 |
| Right PI, | 1.88(1.43,2.78) | 1.70(1.27,2.19) | 0.157 | 1.83(1.53,2.20) | 1.81(1.46,2.15) | 0.901 |
| Left PSV, cm/s | 43.71(35.16,55.61) | 42.54(31.70,51.57) | 0.453 | 42.73(36.94,51.99) | 43.94(33.26,59.99) | 0.875 |
| Right PSV, cm/s | 41.88(33.42,53.72) | 43.96(33.70,56.14) | 0.357 | 41.98(32.25,51.91) | 48.15(43.21,57.18) | 0.003 |
| Left EDV, cm/s | 11.21(7.43,14.62) | 12.47(6.03,19.30) | 0.522 | 14.04 (5.81) | 16.40 (7.33) | 0.134 |
| Right EDV, cm/s | 9.85(7.65,14.30) | 12.67(8.00,20.73) | 0.067 | 9.80(6.86,13.80) | 14.09(12.54,21.50) | <0.001 |

Supplementary Table 3. Comparison of ultrasound vascular tension parameters of young and the middle and elderly group before and after exercise. Note: Normally distributed variables are presented as mean (SD); non-normally distributed variables are presented as median (IQR).

| characteristic | youth group (n=51) | middle and elderly group (n=23) | P |
| --- | --- | --- | --- |
| HR, bpm | 110(100,125) | 112(97,122) | 0.643 |
| CO, L/min | 4.97(3.57,6.11) | 4.77(3.42,5.77) | 0.881 |
| Left RI, | 0.70 (0.14) | 0.69 (0.14) | 0.884 |
| Right RI, | 0.70 (0.11) | 0.75 (0.12) | 0.046 |
| Left PI, | 1.70(1.27,2.19) | 1.72(1.42,1.89) | 0.205 |
| Right PI, | 1.70(1.27,2.19) | 1.81(1.46,2.15) | 0.458 |
| Left PSV, cm/s | 42.54(31.70,51.57) | 43.94(33.26,59.99) | 0.351 |
| Right PSV, cm/s | 43.96(33.70,56.14) | 48.15(43.21,57.18) | 0.209 |
| Left EDV, cm/s | 12.47(6.03,19.30) | 15.50(12.30,21.15) | 0.037 |
| Right EDV, cm/s | 12.67(8.00,20.73) | 14.09(12.54,21.50) | 0.070 |

Supplementary Table 4. Comparison of ultrasound vascular tension parameters of young and the middle and elderly group after exercise. Normally distributed variables are presented as mean (SD); non-normally distributed variables are presented as median (IQR).
